# Massive Pulmonary Embolism Masked as Asthma Exacerbation

**DOI:** 10.7759/cureus.78829

**Published:** 2025-02-10

**Authors:** Nadia Yar, Eric Tran, Lorraine Mukona, Guinda St. Fleur, Maro Valladares, Kim Nguyen, Marai Roque, Huyen Tran

**Affiliations:** 1 Medical School, Chino Valley Medical Center, Chino, USA; 2 Internal Medicine, Chino Valley Medical Center, Chino, USA; 3 Family Medicine, Chino Valley Medical Center, Chino, USA

**Keywords:** asthma, exacerbation, pe, pulmonary embolism, shortness of breath

## Abstract

This case report presents the case of a 41-year-old Caucasian male with no significant past medical history who presented to the emergency department (ED) due to altered mental status. The patient used his brother’s inhaler due to shortness of breath (SOB) and wheezing without any improvement. Later the patient was brought to the ED via Emergency Medical Services (EMS) and was intubated due to severe respiratory acidosis. Although the patient showed some features of pulmonary embolism (PE) on arrival to the ED, PE was not the first differential diagnosis. This case underscores the challenge of recognizing, diagnosing, and managing the early stages of PE in clinical practice, emphasizing the importance of quick recognition and appropriate treatment to improve patient outcomes.

## Introduction

Pulmonary embolism (PE) is a potentially fatal cardiovascular emergency that occurs when a thrombus, typically originating from the deep veins of the lower extremities, embolizes the pulmonary arteries, leading to vascular obstruction, impaired gas exchange, and right ventricular strain. The clinical presentation of PE is notoriously variable, often mimicking more common respiratory conditions such as bronchial asthma or pneumonia. This diagnostic ambiguity presents a significant challenge for clinicians, particularly in patients without identifiable risk factors for venous thromboembolism (VTE). Shortness of breath (SOB) and wheezing are hallmark symptoms of bronchial asthma, a chronic inflammatory disorder of the airways characterized by episodic bronchoconstriction, airway hyperreactivity, and mucus hypersecretion [1]. However, these symptoms can also occur in PE due to the release of vasoactive and inflammatory mediators, such as thromboxane A2 and serotonin, which induce bronchoconstriction.

Studies have demonstrated that pulmonary emboli can precipitate bronchospasm via vagal reflex mechanisms, leading to an asthma-like presentation even in individuals without a prior history of reactive airway disease. This phenomenon further complicates the diagnostic process, particularly in cases where hypoxia and wheezing prompt empiric treatment for asthma, delaying definitive imaging for PE [2]. The overlapping symptomatology of PE, asthma, and pneumonia - including dyspnea, pleuritic chest pain, tachypnea, and cough - necessitates a systematic diagnostic approach. Asthma is typically a clinical diagnosis supported by pulmonary function tests, whereas pneumonia is often identified through radiographic evidence of infiltrates and laboratory markers of infection. In contrast, PE lacks a pathognomonic clinical sign, and its diagnosis is contingent on risk stratification tools (e.g., Wells score, Geneva score), D-dimer testing, and definitive imaging, such as computed tomography pulmonary angiography (CTPA) or ventilation-perfusion (V/Q) scanning.

The challenge of distinguishing PE from these respiratory conditions is further compounded by the presence of "atypical PE presentations" in patients without classic predisposing factors such as recent surgery, prolonged immobility, malignancy, or known thrombophilia. In such cases, reliance on traditional clinical suspicion models may lead to missed or delayed diagnoses, particularly when the patient’s initial presentation strongly suggests an alternative respiratory pathology.

This case report details a 41-year-old Caucasian male with no significant past medical history, who presented with acute onset of shortness of breath, wheezing, and respiratory distress. Initially diagnosed and treated as a case of acute severe asthma, the patient was later found to have a sub-massive pulmonary embolism upon further workup. This case highlights the critical importance of maintaining a broad differential diagnosis in patients presenting with unexplained dyspnea and wheezing, even in the absence of traditional PE risk factors. Furthermore, it underscores the need for early consideration of PE in cases where empiric asthma treatment fails to yield expected clinical improvement.

## Case presentation

A 41-year-old Caucasian male with a past medical history of a previous smoker of 1 pack a day for the last 20 years presented to the emergency department (ED) via emergency medical services (EMS) on 01/12/24 due to altered mental status (AMS). On arrival at the ED at 07:45 a.m., the patient was found to be hypoxic, pulling at his non-rebreather mask, agitated, and unable to provide any verbal history. An initial arterial blood gas (ABG) test was done (Table 1) and shortly after, the patient was given 2 mg lorazepam IV for agitation.

**Table 1:** Initial ABG ABG: arterial blood gas; pCO2: partial pressure of carbon dioxide; pO2: partial pressure of oxygen; HCO3: bicarbonate; O2: oxygen; Pt Temp: patient temperature; Sat: saturation

ABG Parameter	Value
ABG pH	7.21
ABG pCO2 at Pt Temp	51.1 mmHg
ABG pO2 at Pt Temp	66.9 mmHg
ABG HCO3	19.8 mEq/L
ABG O2 Sat (Measured)	85.5%
ABG O2 Sat (Calculated)	86.4%
ABG Base Excess	-8.6 mmol/L
ABG Carboxyhemoglobin	0.3%
ABG Methemoglobin	0.7%

**Figure 1:**
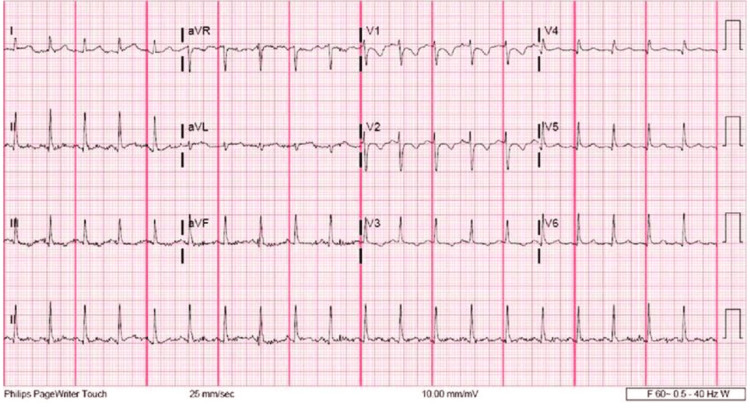
Initial EKG

The patient's father, who was at his bedside, provided the patient’s history; the patient works at an epoxy flooring company and started coughing when he came home at night on 01/10/24. Although the patient did not have a history of asthma, he used his brother’s inhaler due to SOB and wheezing without any improvement. The patient had a history of smoking one pack a day for the last 20 years. He did not drink alcohol or use illicit drugs. He had not received COVID-19 or flu vaccination. Physical examination at ED was remarkable with bilateral 4 mm pupil, tachypneic poor air movement, decreased breath sounds, and mottled bilateral lower extremities. Rectal temperature was 106F. EKG showed tachycardia with a rate of 157 bpm (Figure 1).

**Figure 2:**
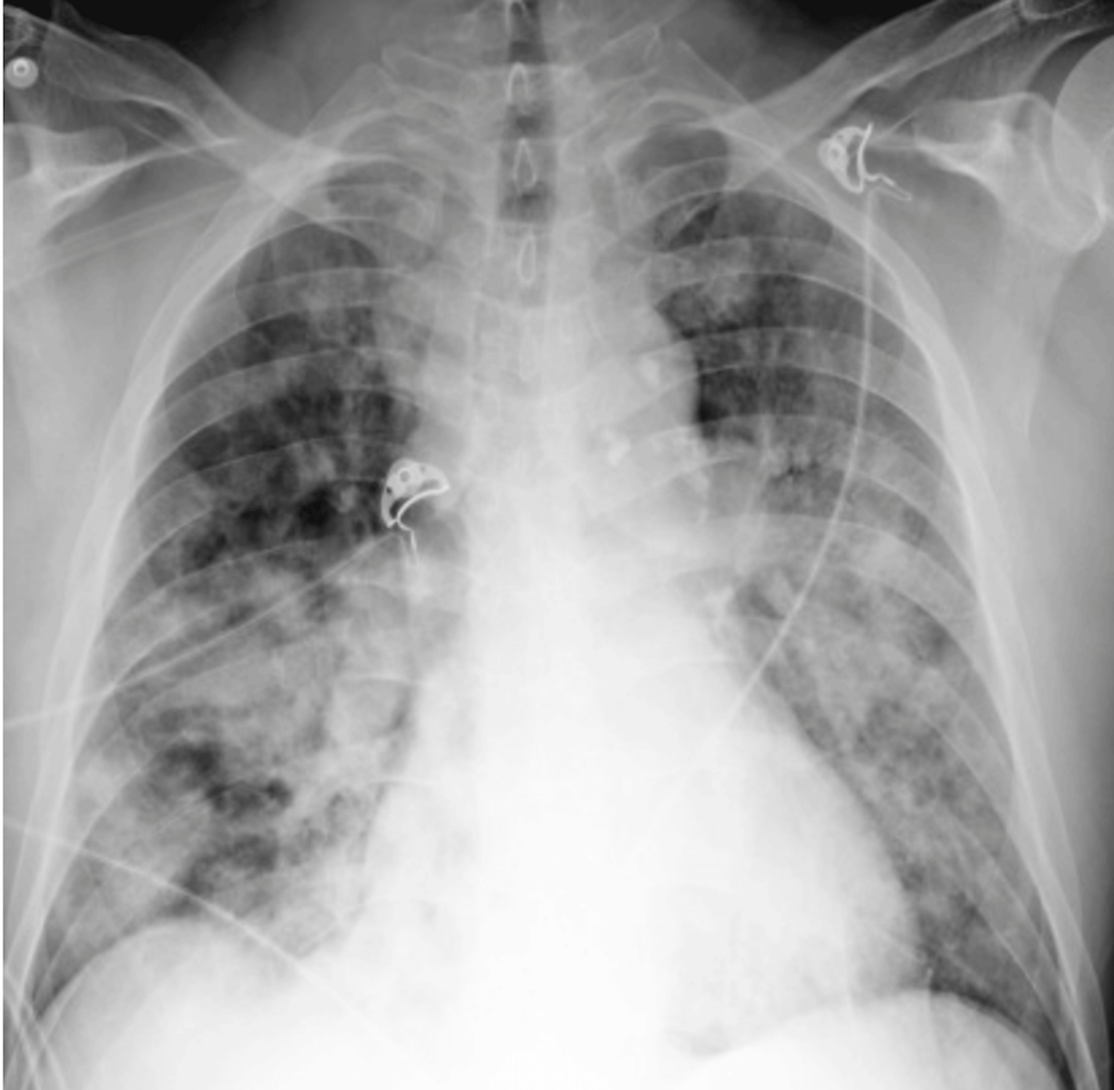
Chest X-ray on admission

Chest X-ray demonstrated multifocal opacities and increased vascularity concern for both pulmonary edema and pneumonia (Figure 2).

**Figure 3:**
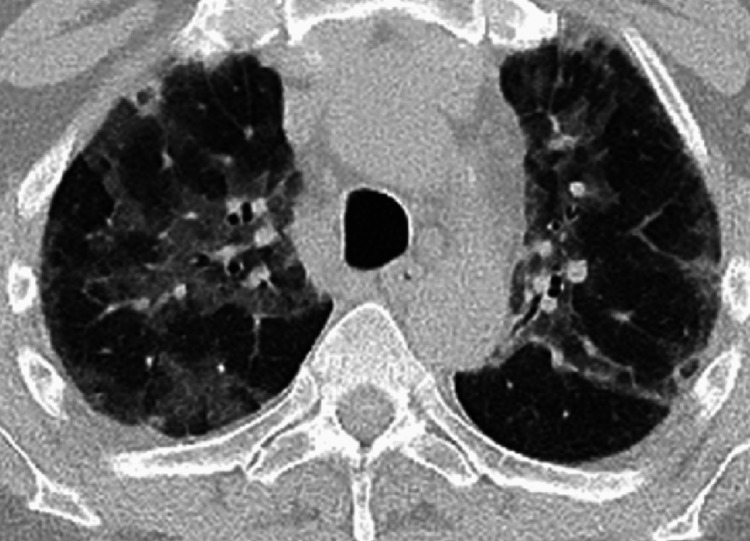
CT Chest without contrast

The patient was placed on a bilevel positive airway pressure (BiPAP) machine for hypoxia, given 500 cc IV bolus, rectal acetaminophen for fever, and vancomycin and piperacillin with tazobactam for possible pneumonia. At 9:00 a.m., the patient remained severely altered and hypoxic with an oxygen saturation of 70% on BiPAP, and a decision was made for emergent intubation. At this time, the patient was tachycardic at 157-160 bpm, and hypertensive with blood pressure (BP) at 166/123 mmHg. Intubation was successful and propofol was started. Hypoxia improved with oxygen saturation of 99% on 100% fraction of inspired oxygen (FiO2). However, since the patient remained hypertensive, he was suspected of having experienced hypertensive pulmonary edema in the setting of sepsis. At 11:00 a.m., CT chest without contrast showed pulmonary edema and patchy infiltrates but no other acute finding in the chest or the abdomen (Figure 3).

Physical examination was remarkable for central cyanosis. Repeat blood gas showed improving pCO2 of 42 mmHg and pO2 of 100 mmHg. However, peripheral oxygen saturation (SpO2) showed 92% on 100% ventilator, which indicated the patient had difficulty being oxygenated. Given those factors, the PE was also considered for differential diagnosis. However, due to renal dysfunction with an increased creatinine of 3, the patient was not a candidate for CT with contrast, which plays a big role in the diagnosis of PE. In addition to active GI bleeding with hematemesis in the orogastric tube (OG tube), the patient was also anuric despite receiving fluid resuscitation and diuretics. The patient was experiencing multiorgan failure, rhabdomyolysis with creatine kinase of more than 7,000 U/L, and cardiac dysfunction. The patient remained severely hyperthermic and was provided 500 cc IV cold fluid, a cooling blanket, ice packs on the axilla, and cool AC. His influenza test came out positive. Repeated vital signs showed an improved heart rate of 135 bpm and BP of 126/72 mmHg.

At 2:05 p.m., the patient was transferred to the ICU, and at 2:11 p.m. code blue was called. After the first round of CPR, the patient regained pulses and was placed on norepinephrine through the peripheral line. Central line placement into the internal jugular vein was attempted, however, during the procedure, the patient became more bradycardic without any pulse, and a second round of CPR was initiated. The patient underwent a total of three rounds of CPR and epinephrine** **and eventually regained pulses on all three occasions after CPR. The patient also had pulseless electrical activity (PEA)** **with an episode of ventricular tachycardia that required defibrillation and was given amiodarone, bicarb, and calcium gluconate. The patient was continued on norepinephrine as well as phenylphrine. Ultimately, at 2:55 p.m., the patient was pronounced deceased.

## Discussion

Pulmonary embolism is usually not the first differential diagnosis made when a patient presents with SOB and wheezing, especially when the patient does not have any previous medical history of deep venous thrombosis (DVT)** **or blood clots. It is challenging to diagnose PE since there is a diverse range of clinical presentations [2]. Some tests are good for ruling in pulmonary embolism, such as helical CT, and some tests are good for ruling out pulmonary embolism, such as D-dimer. However, even with the appropriate use of a combination of noninvasive tests, it is often not possible to definitively diagnose or exclude pulmonary embolism at the initial presentation [3].

This case showed several clinical presentations that correlate with PE: tachypnea, tachycardia, decreased breath sounds, and fever at the initial presentation. The initial findings of the patient's tachycardia with a heart rate of 135 bpm, a D-dimer of 4060 ng/mL, and an elevated troponin of 354 ng/L also correlate with the suspicion of PE. D-dimer is a product that forms when blood clots break down. High levels of D-dimer can indicate that clots are forming and breaking down somewhere in the body, possibly in the lungs, which may suggest a pulmonary embolism (PE). However, those presentations are not specific to PE. The chest X-ray showed mild diffuse interstitial edema and moderate pulmonary vascular congestion suggesting more towards cardiogenic lung edema. If the X-ray had shown a normal or negative chest X-ray and the result of arterial blood gas was low like this patient (Table 1), suspicion of PE would have been more obvious.

The range of normal values varies among laboratories. In general, normal values for acidity (рΗ), the partial pressure of carbon dioxide (PCO2) and bicarbonate concentration (HCO3) are as follows рΗ - 7.35 to 7.45, PCO2 - 35 to 45 mmHg (4.7 to 6 kPa), HCO3 - 21 to 27 mEq/L [4]. The most common EKG findings for PE is S1Q3T3, and the patient's EKG findings were borderline T wave abnormalities in the inferior leads which could raise the suspicion of PE. However, it was not a PE-specific finding. Additionally, wheezing was recently reported as occurring in some patients suspected of having a PE concurrent with asthma likely secondary to bronchoconstriction [5,6]. One of the crucial medical treatments for PE is to initiate anticoagulation prior to confirming the diagnosis of PE. This was one of the challenges in that the patient could not receive this medication since he was experiencing active GI bleeding from his OG tube inserted after intubation on admission. Although inferior vena cava filters (IVCfilters) are particularly useful in patients who cannot receive blood thinners due to the risk of bleeding, they are generally considered a temporary solution and are often removed. A CTPA could be done once PE was suspected, however, the patient's creatinine level was 3 and anuric, therefore, it was not possible to proceed with a CTPA.

There were many challenges in identifying PE for this case. First, the patient’s history was not easy to obtain since the patient’s mental status had been altered. The most common findings of PE are dyspnea, pleuritic chest pain, cough, calf or thigh pain and/or swelling, wheezing, and hemoptysis. Some of those symptoms were gathered from his family members, but obtaining the patient's medical history directly from him would have been more beneficial. Also, point-of-care ultrasound (POCUS) is very useful and could have been helpful in aiding in a quicker diagnosis due to no CTPA. It is quick and can be used at the bedside. Multiorgan rather than single-organ POCUS can be of aid in ruling out PE in the critically ill and help select patients for CTPA [7]. While useful, it is important to note that ultrasound is not diagnostic in PE and can only aid in showing possible right heart strain. An additional challenge was the patient arriving in a severely critical condition at the ED, leading to significant limitations in conducting essential tests to confirm PE.

## Conclusions

This case highlighted a number of the significant challenges in diagnosing PE. There are many variable clinical presentations of PE, and they are often nonspecific, making the diagnosis challenging. Once PE is suspected, initiating anticoagulation plays a big role in optimizing a patient’s positive outcome. POCUS might show right ventricular strain, indicating the suspicion of PE and allowing for prompt treatment. PE can be life-threatening and sometimes fatal. Therefore, keeping PE in your differentials when the patient presents with SOB, wheezing, and low ABG is the key to prompt recognition of PE and initiating immediate treatment.
